# Thermodynamic Analysis of n-Nonadecane (C_19_H_40_)/1-Octadecanol (C_18_H_37_OH) Blends

**DOI:** 10.3390/molecules29122722

**Published:** 2024-06-07

**Authors:** Wentao Guo, Yi Xing, Wei Wen, Wei Su, Changjiang Hou, Guotao Li, Lyumeng Ye

**Affiliations:** 1 School of Energy and Environmental Engineering, University of Science and Technology Beijing, Beijing 100083, China; wentao_guo@ustb.edu.cn (W.G.); xingyi@ustb.edu.cn (Y.X.); 2 Group Strategic Research Institute, HBIS Group Co., Ltd., Shijiazhuang 050023, China; houchangjiang@hbisco.com (C.H.); liguotao@hbisco.com (G.L.); 3 Guangdong Province Engineering Laboratory for Air Pollution Control, South China Institute of Environmental Sciences, The Ministry of Ecology and Environment of People’s Republic of China, Guangzhou 510655, China; yelvmeng@scies.org

**Keywords:** molecules, crystal structure, crystallisation, binary system, phase transition, thermodynamics, long-chain hydrocarbons

## Abstract

The article delves into the intricate phase transitions of 1-Octadecanol and n-Nonadecane within a binary system, unveiling dynamic structural changes under varying conditions. Through Fourier transform infrared (FTIR) analysis, specific molecular vibrations were identified, shedding light on the molecular composition and interactions. The study highlights the challenges in detecting subtle phase transitions and emphasises the individuality of molecular behaviours in closely related compounds. The findings underscore the complexity of phase transitions in binary systems and advocate for a nuanced approach to studying molecular structures and behaviours.

## 1. Introduction

### 1.1. Research Backgrounds

The transition of land plants from an aquatic to a terrestrial lifestyle was accompanied by the development of a novel structure known as the cuticle [1,2]. This lipophilic layer, composed of cutin and cuticular waxes, had the primary purpose of limiting water loss that was not caused by stomata by acting as a physical barrier between the surface of the plant and the environment outside of the plant [3,4,5]. Forming a cuticular wall is one of the primary adaptation strategies that allow plants to survive and thrive in water-restricted terrestrial environments [6,7,8]. The cuticle protects the plant from mechanical rupture or injury, poisonous chemicals, and ultraviolet radiation [5,9,10]. It serves as the primary barrier between the aerial surface of plants and the external environment. The cuticle also plays essential roles in the processes of growth and development, such as avoiding epidermal fusion by establishing appropriate organ borders [2,11] and maintaining phytohormone homeostasis [12,13]. These are only two examples of these roles. It is well known that the cuticle and the components that make up the cuticle perform essential roles as signalling molecules for pathogens that attack plants and the plants themselves [14]. It also plays a significant function in fruits, affecting quality, defence, and shelf life after harvest [2]. In fruits, the cuticle can affect water retention, hardness [10,13], and reactions to both biotic and abiotic stressors.

There is much research on plant wax globally, and the understanding of its composition is also more profound. Plant epidermal wax is a mixture of hydrophobic organic substances that appear as a whitish-grey or greenish-grey frost covering the outer layers of epidermal plant cells. Waxes are not unique to plant leaves, but studies on waxes have been mainly focused on plant leaves. The chemical composition of waxes is mixed, and the chemical substances in waxes can be extracted effectively with organic solvents such as trichloromethane (CHCl_3_). Modern studies primarily rely on gas chromatography–mass spectrometry (GC–MS) and nuclear magnetic resonance (NMR) techniques to identify wax components, and more than 100 compounds have been identified. The chemical features of waxes mainly include aliphatic compounds and cyclic compounds. Aliphatic compounds include long-chain (larger than C_18_) and ultra-long-chain (C_24_–C_36_) fatty acids, alkanes, alcohols, aldehydes, ketones, and esters, with different odd–even state advantages. Ring compounds contain phenols, terpenoids, and flavonoids, and some of them are small molecular secondary metabolites of plants. The form and content of plant cuticle vary greatly across and within species and include plate-, needle-, and pillar-shaped wax crystals. This variation is to be anticipated given the diversity of plants, the ecosystems they occupy, and the unique life histories of individual plants [15]. In certain species, the composition of the cuticular wax is known to change with increasing depth, resulting in the formation of layers that may be chemically differentiated [16]. In addition, the cuticle has been demonstrated to have a crucial role in both the development and the pathophysiology of diseases [16,17,18,19,20,21,22]. It should not come as a surprise, therefore, that interest in the content, structure, and physiology of cuticles is growing [5,22,23,24]. A better understanding of the relationship between the structure and the chemical composition of cuticle waxes (and its relation to Inter- and Intra-Chain Interactions in Mixtures of Long-Chain Hydrocarbons) is essential for increasing agricultural yields. This is because it will further our knowledge of how plants regulate water balance and inform the application of nutrition (foliar feeds) and pesticides, which will lead to improved formulation strategies for agrochemicals. Therefore, a knowledge of the inter- and intra-chain interactions in mixtures of long-chain hydrocarbons, in connection to plant cuticular waxes, can give a leading niche to the fulfilment of the protection and future investigations into a more effective cuticular protection component.

According to recent research, the major transpiration barrier is formed by intra-cuticular waxes, and the contribution of the epi-cuticular waxes as a transpiration barrier varies on the makeup of the cuticle of each species. And intra-cuticular wax acts as a transportational barrier [25].

### 1.2. Research Approaching

Extensive research conducted in the past decade has primarily focused on two major chemical families: n-alkanes (C_n_H_2n+2_) and 1-alkanols (C_n_H_2n+1_OH), examining their structural and thermodynamic aspects. N-alkanes, which are commonly present in petroleum waxes, are significant due to their blending properties and serve as effective models for understanding complex substances like polymers and bio-membranes. Previous studies have investigated a total of 19 binary systems containing both odd and even n-alkanes ranging from 8 to 28 carbon atoms [26,27,28,29,30,31,32]. Experimental phase diagrams have revealed that complete miscibility is not observed at lower temperatures, especially within the solid-ordered phase region, despite the matching of crystal symmetries in pure components (as seen in both odd–odd and even–even alkane systems). Instead, the mixing of substances stabilises various metastable crystalline structures, typically seen in longer alkanes starting from C_25_H_52_ [33].

Alkanols, which are simple substituted hydrocarbons, consist of an aliphatic chain with a hydrogen atom replaced by a single -OH group at one end. Previous researchers have examined pure alkanols ranging from 1-undecanol to 1-eicosanol, as well as 10 binary systems containing components with 15–20 carbon atoms. Studies on n-alkane systems have shown that mixing can stabilise forms that are only metastable for the pure components, especially noticeable in even–even systems. For instance, even–even systems made up of isostructural components do not exhibit continuous miscibility; instead, mixing stabilises a form that is stable in odd alkanols at low temperature.

To ensure global compatibility within organic families, this study expands its scope to investigate binary systems consisting of n-alkanes and 1-alkanols. The main objective is to explore the solid-state compatibility and potential applications of the resulting blends. Previous experimental and thermodynamic studies have primarily focused on the solid–liquid equilibria of binary systems comprising even n-alkanes (ranging from octane to hexadecane) and 1-alkanols (from 1-butanol to 1-eicosanol) [33]. This research specifically aims to experimentally determine equilibrium phase diagrams, analyse phase transitions, and investigate the thermodynamic properties when n-Nonadecane (C_19_H_40_) and 1-Octadecanol (C_18_H_37_OH) interact.

## 2. Results

To delve deeper into the complexities of the 1-Octadecanol and n-Nonadecane binary system, a fascinating sequence of phase transitions that reveal the intricate nature of these compounds was observed. For 1-Octadecanol, the transition begins in a liquid state, before transitioning to an R’IV phase, and finally stabilizing in a monoclinic structure. This progression demonstrates the compound’s dynamic structural changes under varying conditions.

Similarly, n-Nonadecane exhibits its own unique transformation path. It starts from a liquid state and then moves into the R’I phase. Following this, it settles into an orthorhombic structure. This pathway underscores the distinct molecular behaviours of n-Nonadecane compared to 1-Octadecanol, even though they are part of the same binary system.

A particularly intriguing aspect of this study is the transition of 1-Octadecanol from the γ-phase to the rotator phase. This specific transition phase, which is of significant scientific interest, proves elusive when attempting to detect it using Differential Scanning Calorimetry (DSC). The challenge in detecting this transition lies in the extremely subtle nature of the signal that this phase change generates. It is a testament to the sensitivity required in measuring techniques and the need for precise instrumentation in the study of molecular phase transitions.

Moreover, the differences in the transition pathways of 1-Octadecanol and n-Nonadecane, despite being components of the same binary system, highlight the complexity and individuality of molecular structures and behaviours. These findings have broader implications for the study of phase transitions in binary systems, suggesting that even closely related compounds can exhibit markedly different behaviours under similar conditions. This complexity necessitates a more nuanced approach to the study of phase transitions in such systems, with a focus on the individual characteristics of each component as well as their interactions.

Drawing upon preceding research [33,34], the Fourier transform infrared (FTIR) analysis yielded valuable insights. Manifesting as a peak around the wavenumber 750 cm^−1^, the out-of-plane bending of the O-H bond was discerned. The peak encompassing 1000 cm^−1^ signified the stretching vibration of the C-O bond, while the peak at 1500 cm^−1^ denoted the bending vibration of the C-H bond. The distinctive double peak discerned around 3000 cm^−1^ was attributed to the saturated C-H stretching vibration absorption. Notably, an inconspicuous yet broad peak around 3300 cm^−1^ was identified as the O-H stretching vibration indicative of intermolecular hydrogen bonds. These nuanced analytical findings contribute significantly to our understanding of the molecular dynamics and structural intricacies within this binary system.

### 2.1. DSC Diagrams

#### 2.1.1. Cooling Rate at 1 °C/min

**Figure 1:**
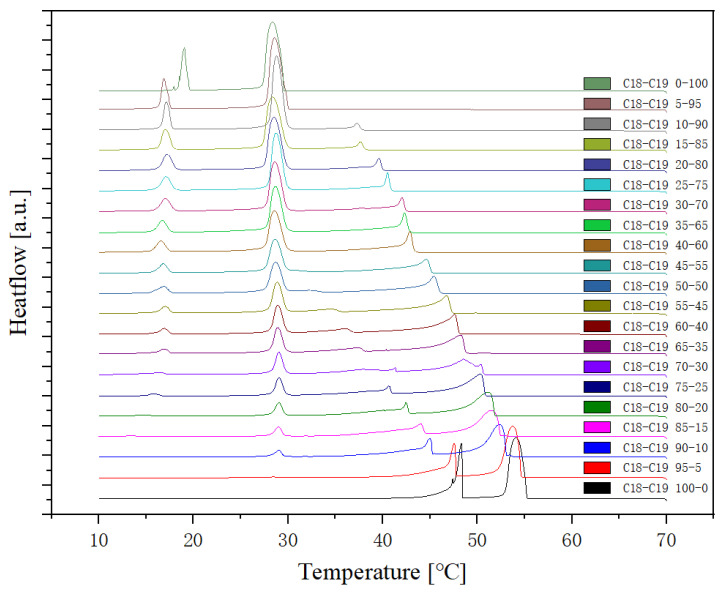
Cooling Phase diagram at 1 °C/min (Cycle 1).

The cooling Differential Scanning Calorimetry (DSC) diagram for heating and cooling cycle 1, conducted at a rate of 1 °C/min (Figure 1), reveals a nuanced profile with a maximum of four peaks and a minimum of two peaks. The dynamic nature of the peaks is closely tied to the evolving composition during the process. Notably, the rightmost peak signifies the crystallisation of 1-Octadecanol from the liquid phase into a rotator phase. Following this, the subsequent peak denotes the transition of 1-Octadecanol from the rotator phase to an ordered phase. On the left side of the spectrum, attention is drawn to the two peaks related to n-Nonadecane. The rightward of these depicts the crystallisation of n-Nonadecane from the liquid phase into a rotator phase, while the leftward peak marks the transition of the rotator-phase-n-Nonadecane into an ordered phase. The shifting positions of these peaks as the composition undergoes alterations provide valuable insights into the intricate thermodynamic transformations occurring within the substance, offering a comprehensive understanding of its phase transitions and crystallisation behaviour during the specified heating and cooling cycle [35,36].

**Figure 2:**
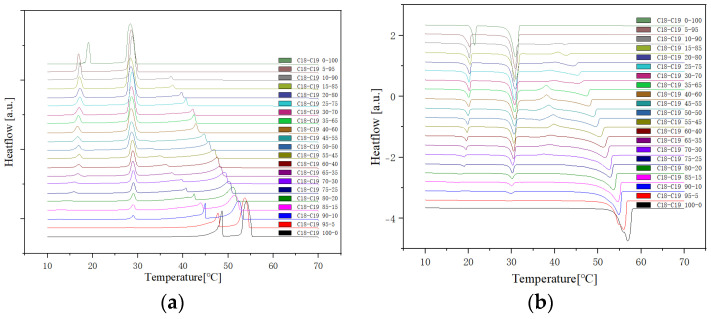
Cooling (**a**) and Heating (**b**) Phase diagram at 1 °C/min (Cycle 1).

**Figure 3:**
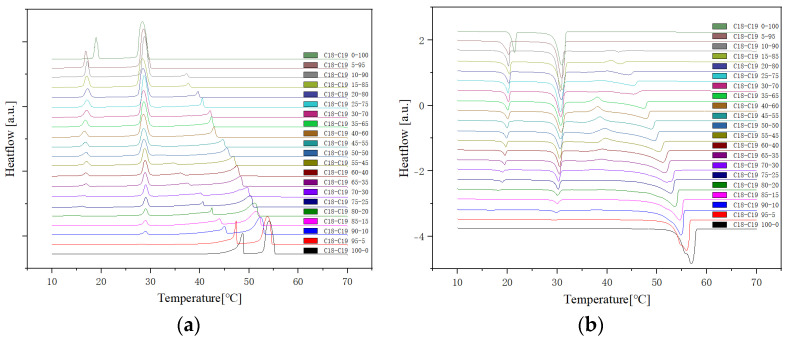
Cooling (**a**) and Heating (**b**) Phase diagram at 1 °C/min (Cycle 2).

**Figure 4:**
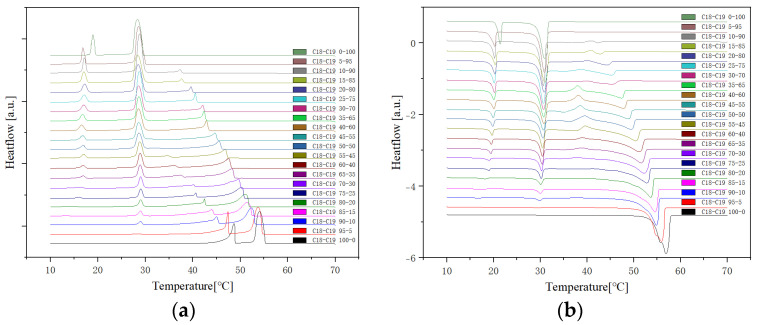
Cooling (**a**) and Heating (**b**) Phase diagram at 1 °C/min (Cycle 3).

Presented below (Figure 2, Figure 3 and Figure 4) are comprehensive Differential Scanning Calorimetry (DSC) diagrams recorded at a scan rate of 1 degree Celsius per minute during three cycles (1 °C/min). The upper trio of diagrams illustrates the cooling phase, providing a detailed exploration of the thermal transitions occurring in the material under decreasing temperatures. Conversely, the subsequent set of diagrams captures the heating phase, shedding light on the reverse process as the material warms up. This collection of DSC diagrams serves as a meticulous representation of the thermal behaviour, elucidating key transformations and transitions that unfold within the substance across varying temperature ranges. The cooling diagrams offer insights into the solidification and crystallisation processes, while the heating diagrams unveil the corresponding melting events and other thermal phenomena, collectively contributing to a comprehensive understanding of the material’s thermal characteristics.

The analysis of the thermal behaviour presented in the comparison of the three cycles, each conducted at a constant cooling and heating rate of 1 °C per minute, reveals a remarkable consistency. In observing the Specific DSC diagram provided below, the cooling and heating segments are visually distinguishable. The blue portion signifies the cooling procedure of the sample, while the red part corresponds to the heating phase. Notably, despite the dynamic transitions in temperature, the absence of discernible differences in the cooling and heating rates across the three cycles indicates an exceptional level of stability exhibited by the samples.

**Figure 5:**
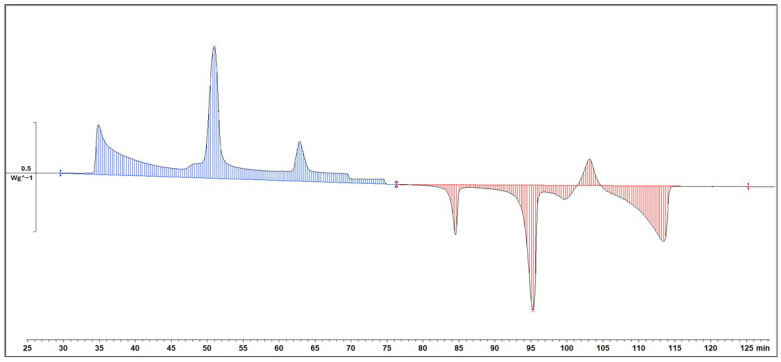
An example of DSC diagram in STAR^e^ software (pure 1-Octdecanol) Note: (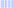) Exothermic reaction phases of 1-Octadecanol, (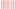) Endothermic reaction phases. The horizontal coordinate is the time, and the vertical coordinate is the weight difference of the sample.

Delving deeper into the intricacies of the analysis, attention is drawn to the significance of the peaks within the DSC diagram. These peaks encapsulate critical information about the sample’s behaviour during phase transitions. By integrating the area under each peak, we gain valuable insights into the enthalpy associated with each specific phase transition. This approach allows for a nuanced understanding of the energy changes occurring within the sample, providing a comprehensive perspective on its thermal properties. In essence, the Specific DSC diagram serves as a visual representation of the stability and thermodynamic characteristics of the material under investigation, elucidating the subtleties of its responses to varying temperature conditions. For instance, the Differential Scanning Calorimetry (DSC) profile of pure 1-Octadecanol, as depicted in Figure 5 using STAR^e^ software (version 16.40), was obtained under specific experimental conditions. These conditions included a heating rate of 1 °C per minute and a corresponding cooling rate. Within this diagram, the blue segment illustrates the exothermic reaction phase of 1-Octadecanol, whereas the red segment delineates its endothermic reaction phase. Two tables of phase transition temperature and enthalpy value of each peak (Table 1 and Table 2) were obtained by concluding all DSC diagrams.

#### 2.1.2. Cooling Rate at 0.75 °C/min and 0.5 °C/min

**Figure 6:**
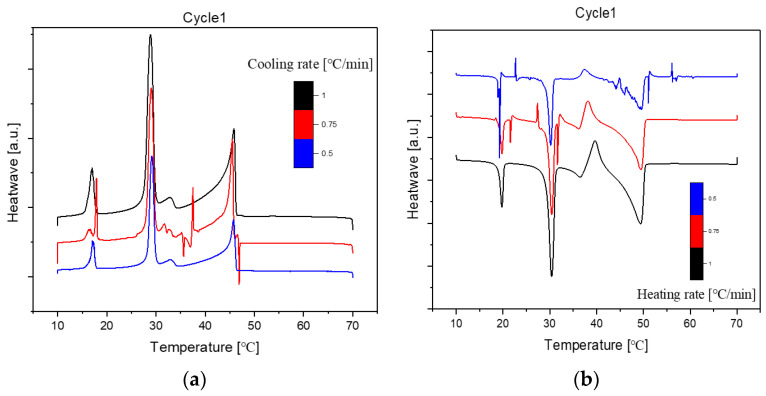
Differential Scanning Calorimetry (DSC) diagram at cooling (**a**)/heating (**b**) different rates (1, 0.75, 0.5 °C/min, Cycle 1). Note: (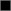): 1, (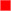): 0.75, (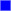): 0.5.

**Figure 7:**
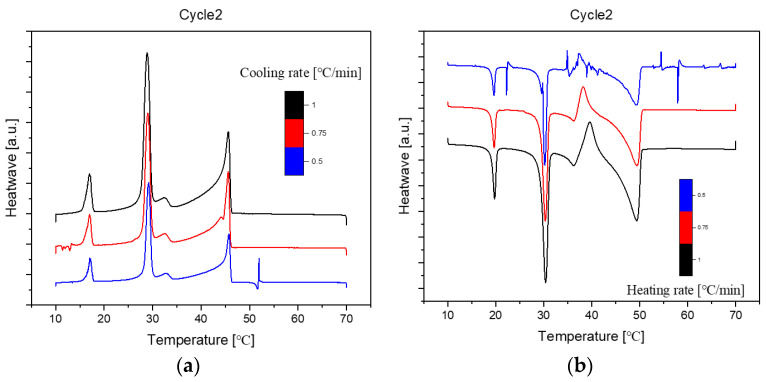
Differential Scanning Calorimetry (DSC) diagram at cooling (**a**)/heating (**b**) different rates (1, 0.75, 0.5 °C/min, Cycle 2). Note: (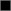): 1, (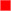): 0.75, (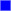): 0.5.

**Figure 8:**
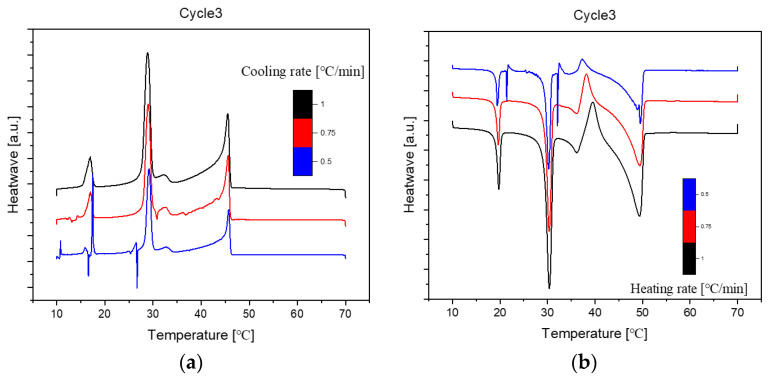
Differential Scanning Calorimetry (DSC) diagram at cooling (**a**)/heating (**b**) different rates (1, 0.75, 0.5 °C/min, Cycle 3). Note: (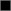): 1, (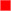): 0.75, (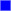): 0.5.

The DSC (Differential Scanning Calorimetry) diagrams depicting various cooling or heating rates for sample No. 11 with a composition of 50% n-Nonadecane and 50% 1-Octadecanol are illustrated through Figure 6, Figure 7 and Figure 8. Notably, the altitude of the peaks exhibited a decreasing trend as the rate of cooling or heating diminished. This phenomenon is attributed to the profound influence of the temperature ramp rate on resolution and sensitivity during sample analysis.

In general, a faster rate of temperature increases results in lower resolution and higher sensitivity, while a slower rate contributes to higher resolution and lower sensitivity. As the ramp rate escalates, the onset temperature of the melt peak remains relatively stable, but the peak top and end temperatures rise, leading to a broader peak shape. Rapid ramp-up processes tend to induce superimposed thermal effects, causing inadequate separation of individual peaks or melts associated with different phases.

Conversely, during the cooling phase, the rate influences the crystallisation behaviour. A swift cooling rate tends to delay crystallisation, yet this method proves advantageous in optimizing product processing. Notably, the overall peak shape at varying rates closely resembled the diagram obtained at a consistent rate of 1 °C/min. This consistency implies that the sample utilised for diverse rate scanning exhibited stability. Consequently, it can be inferred that all samples share a common stability, contributing to a comprehensive understanding of the thermodynamic behaviour and phase transitions in the tested mixtures.

#### 2.1.3. Reproducibility

**Figure 9:**
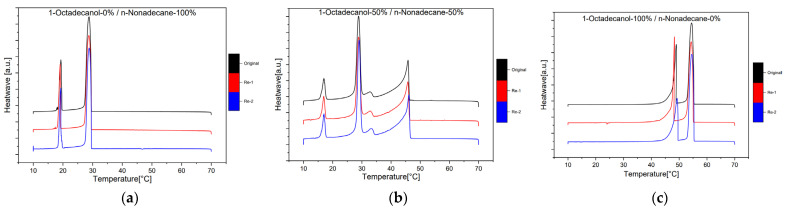
Reproducibility Differential Scanning Calorimetry (DSC) diagram (1 °C/min) with different compositions; (**a**) 1-Octadecanol 0%/n-Nonadecane 100%; (**b**) 1-Octadecanol 50%/n-Nonadecane 50%; (**c**) 1-Octadecanol 100%/n-Nonadecane 0%. Note: (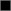): Samples in Table 3, (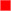): 1st Reproduction, (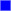): 2nd Reproduction.

The reproducibility of the experiment was a crucial aspect of our investigation, and rigorous measures were taken to ensure the reliability of the results. To assess this, three separate samples were independently run at 1dCpm, each undergoing two additional repetitions. The obtained data were meticulously analysed, and the corresponding chromatograms were compared. The resulting chromatograms, as depicted in Figure 9, revealed a striking similarity in both the shape and height of the peaks across all replicates. In fact, the resemblance was so pronounced that the chromatograms appeared almost identical. This remarkable consistency in the patterns observed strongly indicates that the experiment was highly reproducible. The reliable replication of results not only attests to the robustness of our methodology but also instils confidence in the credibility and accuracy of the findings derived from this experimental setup.

### 2.2. FTIR Spectra

The analysis of seven samples, as outlined in Table 3, involved the utilisation of Fourier transform infrared (FTIR) spectroscopy. These samples, with varying compositions of 1-Octadecanol at 100%, 80%, 60%, 50%, 40%, 20%, and 0%, were selected for a comprehensive investigation.

**Figure 10:**
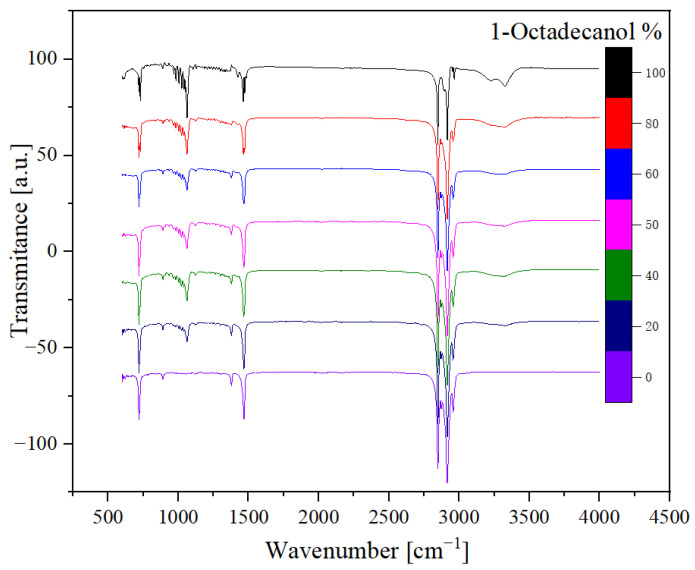
FTIR spectra (Transmittance).

**Figure 11:**
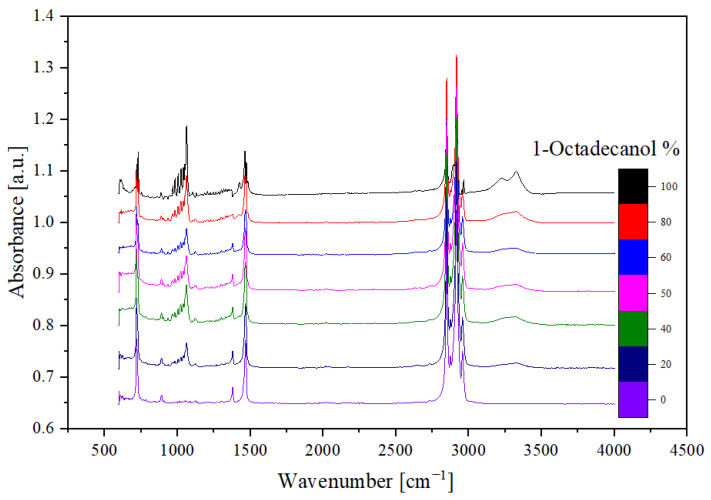
FTIR spectra (Absorbance).

The FTIR spectra obtained from these samples are visually represented below, offering valuable insights into the molecular composition. Figure 10 illustrates a spectrum comparing transmittance and wavenumber, while Figure 11, the derived transformation from Figure 10 through OMNIC, depicts additional spectra comparing absorbance and wavenumber.

In the spectral analysis, distinctive peaks were identified, each corresponding to specific molecular vibrations. FTIR spectra at various concentrations show some major characteristic absorption peaks that correspond to the vibration patterns of different functional groups in n-Nonadecane and 1-Octadecanol blends.

Notably, the O-H stretching vibration: Within the range of approximately 3200–3600 cm^−1^, broad and intense absorption peaks can be observed, corresponding to the O-H bond stretching vibrations in 1-Octadecanol. C-H stretching vibrations: In the range of about 2850–2950 cm^−1^, strong absorption peaks are present, corresponding to the symmetric and asymmetric stretching vibrations of C-H bonds (-CH_2_- and -CH_3_ groups). These peaks primarily originate from the alkyl chains in n-Nonadecane and 1-Octadecanol. C-H bending vibration: Significant absorption peaks appear at around 1465 cm^−1^ and 1375 cm^−1^, associated with the bending vibrations of the C-H bonds. C-O stretching vibration: Within the range of approximately 1050–1150 cm^−1^, a moderate intensity absorption peak can be detected, corresponding to the C-O bond stretching vibration in 1-Octadecanol.

Different mixing ratios of 1-octadecanol and n-nonadecane exhibit variations in absorption peak intensity and peak positions. The absorption intensity of the O-H stretching vibration peak increases with the rising proportion of 1-Octadecanol, while the relative intensity of the C-H stretching vibration peak becomes more pronounced with higher proportions of n-Nonadecane. For instance, samples with 80% and 100% 1-Octadecanol show significant O-H stretching vibration peaks in the 3200–3600 cm^−1^ range, whereas samples with lower concentrations (such as 0% and 20% 1-Octadecanol) do not exhibit notable absorption peaks in this region. The C-H stretching vibration peak is present at all mixing ratios, but its intensity and shape vary according to the mixing ratio, reflecting the relative content of 1-Octadecanol and n-Nonadecane.

Of particular interest was the inconspicuous yet significant wide peak at around 3300 cm⁻¹. This peak was attributed to the O-H stretching vibration associated with intermolecular hydrogen bonds. The presence and characteristics of these peaks provide valuable information about the molecular structure and interactions within the samples, contributing to a comprehensive understanding of the composition and behaviour of 1-Octadecanol in different proportions. This FTIR analysis serves as a crucial tool in unravelling the intricate details of the molecular dynamics of these samples and contributes to the broader understanding of their chemical properties [34,39].

### 2.3. Phase Diagram

#### 2.3.1. Cooling Phase Diagram

**Figure 12:**
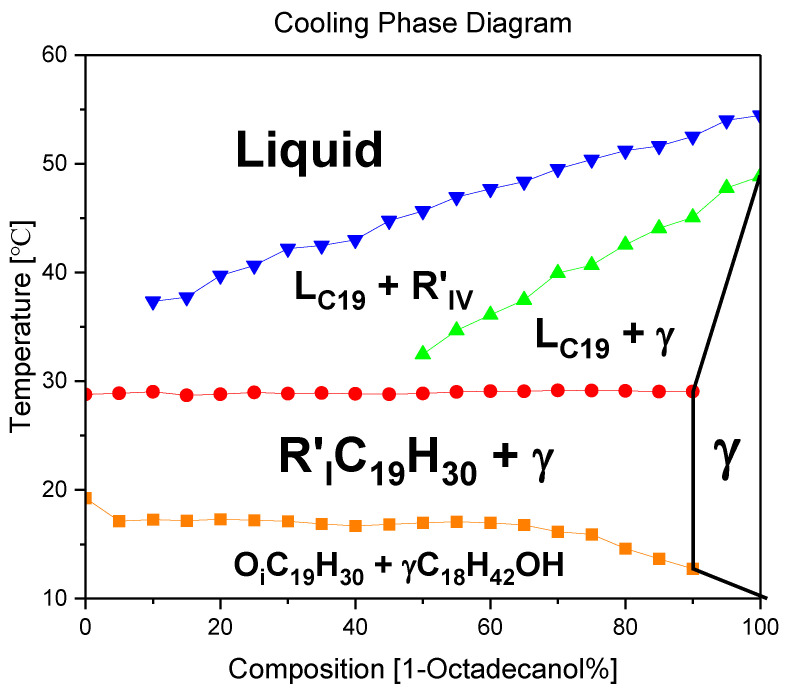
A binary system Phase Diagram of n-Nonadecane and 1-Octadecanol (Cooling) and Phases. Note: (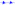) Thermal profile of 1-Octadecanol from the liquid state to the R’IV phase, observed across varied proportions of 1-Octadecanol to n-Nonadecane, (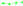) Temperature trajectory of 1-Octadecanol during its phase transition from R’IV to the Monoclinic (γ) phase, (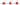) Temperature evolution of n-Nonadecane as it undergoes a phase shift from the liquid state to the R’I phase, (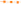) Thermal progression of n-Nonadecane during its transition from the R’I phase to the Orthorhombic (β) phase.

The construction of a binary system phase diagram involved a meticulous analysis of the DSC (Differential Scanning Calorimetry) diagram, where the peak temperatures were systematically summarised. This comprehensive diagram (Figure 12) serves as a visual representation of the intricate phase transitions occurring within the constituent materials. Each segment of the binary system is distinctly depicted, allowing for a nuanced understanding of the thermodynamic behaviour.

Upon careful examination of the phase diagram, a significant observation emerges: the absence of any discernible evidence pointing towards Eutectic or Peritectic reactions. Eutectic reactions typically involve the simultaneous solidification of two or more phases from a liquid, while Peritectic reactions entail the transformation of one solid phase into another. The lack of indicators for these reactions in the diagram underscores the stability and unique characteristics of the binary system under consideration.

In essence, the plotted phase diagram not only elucidates the temperature-dependent transitions within the binary system but also provides crucial insights into the absence of specific reactions that might alter its composition or structure. This analytical approach not only enhances our understanding of the thermodynamic behaviour of the materials involved but also contributes to the broader field of materials science by unravelling the intricate interplay of phases within binary systems.

In the thermal journey from elevated temperatures to lower ones, the dynamic transformations of 1-Octadecanol exhibit a sequence of phases. Initially existing in a liquid state, it undergoes a transition to the R’IV phase, characterised by specific structural arrangements. As the temperature further decreases, the 1-Octadecanol experiences another shift in its molecular organisation, entering the Monoclinic (γ) phase. This progression signifies the intricate interplay of molecular forces and interactions within the substance as it responds to variations in thermal energy.

Conversely, the phase transitions of n-Nonadecane follow a distinct pathway. Originating as a liquid at higher temperatures, it undergoes a transformative shift to the R’I phase, marked by a distinct structural configuration. Subsequently, as the temperature continues to decrease, n-Nonadecane transitions into the Orthorhombic (β) phase, denoting a specific crystalline arrangement of its molecular constituents. This intricate transition between liquid and crystalline phases underscores the nuanced behaviour of n-Nonadecane under varying thermal conditions. In essence, these observations emphasise the sensitivity of these substances to temperature variations, unveiling a rich tapestry of phase transitions governed by molecular intricacies.

According to previous studies, the crystallisation behaviour of n-Nonadecane is influenced by the single layer on the surface, resulting in the formation of a new hexagonal rotating phase (R II), which has not been reported in the n-Nonadecane system [40]. In the microcapsule, n-Nonadecane is first trapped in a new metastable phase, then transitioned to the RII phase, and finally to the most stable orthorhombic phase 4. The emergence of this new metastable phase is mainly due to the formation of R II phases induced by the frozen monolayer on the surface in the microcapsule [41]. 1-Octadecanol (C_18_H_37_OH) presents a γ phase at room temperature, and its cell parameters are a = 9.031 Å, b = 4.959 Å, c = 98.19 Å, and β = 122.41°4 Å. During the heating process, the γ phase is transformed into a monoclinic rotating phase R’IV at a transition temperature of about 329.5 K and an enthalpy of 26.5 kJ/mol [42].

The blue line in the graph represents the temperature profile of 1-Octadecanol as it transitions from the liquid phase to the R’IV phase under varying ratios of 1-Octadecanol to n-Nonadecane. Concurrently, the green line delineates the temperature trajectory of 1-Octadecanol during its phase transition from R’IV to the Monoclinic (γ) phase. Meanwhile, the red line corresponds to the temperature curve of n-Nonadecane as it shifts from the liquid phase to the R’I phase. Lastly, the orange line illustrates the temperature progression of n-Nonadecane as it undergoes a phase transition from the R’I phase to the Orthorhombic (β) phase.

Overall, due to the phase diagram, from high temperature to low temperature, the phase transitions of 1-Octadecanol are Liquid → R’IV → Monoclinic (γ) [35,36,43]; on the other hand, the phase transitions of n-Nonadecane are Liquid → R’I → Orthorhombic (β) [34,37,38,43].

#### 2.3.2. Heating Phase Diagram

**Figure 13:**
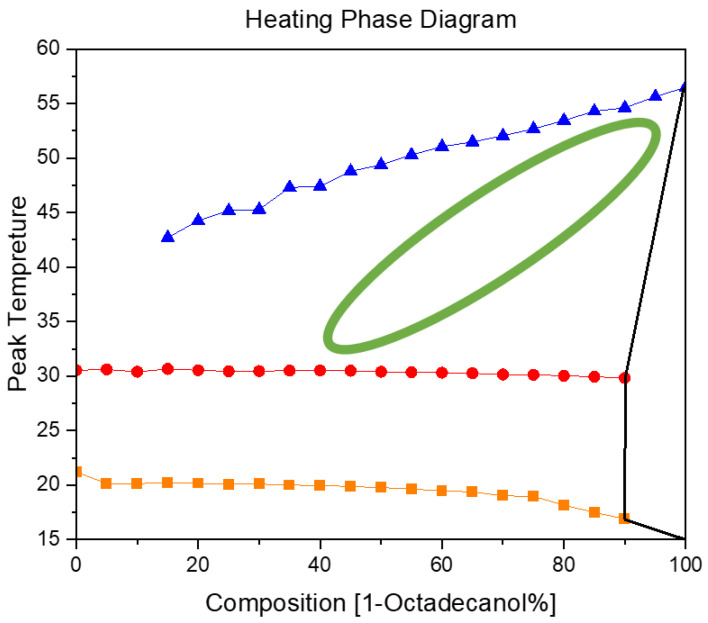
A binary system Phase Diagram of n-Nonadecane and 1-Octadecanol (Heating), Note: (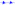) Thermal profile of 1-Octadecanol from the liquid state to the R’IV phase, observed across varied proportions of 1-Octadecanol to n-Nonadecane, (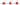) Temperature evolution of n-Nonadecane as it undergoes a phase shift from the liquid state to the R’I phase, (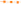) Thermal progression of n-Nonadecane during its transition from the R’I phase to the Orthorhombic (β) phase, (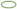) Phase transitions undergone by 1-Octadecanol.

In Figure 13, the blue trajectory delineates the thermal profile of 1-Octadecanol during its phase transition from the liquid state to the R’IV phase, observed across varied proportions of 1-Octadecanol to n-Nonadecane. Simultaneously, the red trajectory signifies the temperature evolution of n-Nonadecane as it undergoes a phase shift from the liquid state to the R’I phase. Furthermore, the orange trajectory graphically represents the thermal progression of n-Nonadecane during its transition from the R’I phase to the Orthorhombic (β) phase [35,36,37,38].

Examining the Cooling and Heating Phase diagram allows for a discerning analysis of the phase transitions undergone by 1-Octadecanol. Notably, the transition from a stable phase to a rotator phase is evident. Intriguingly, during the heating process, this transition seems to dissipate (the region delineated by the green ellipse, comparing with Figure 12), suggesting that the disordered phase is not thermodynamically stable under elevated temperatures. Although a subtle phase transition is observable during heating, its intensity appears insufficient for accurate recognition by Differential Scanning Calorimetry (DSC). This implies that certain alterations in the molecular arrangement or energy state occur, but they might be subtle or transient, eluding detection by the precision of the DSC technique. This nuanced understanding of the phase behaviour of 1-Octadecanol sheds light on the intricacies of its thermodynamic stability and the sensitivity required in experimental methodologies to capture subtle transformations.

**Figure 14:**
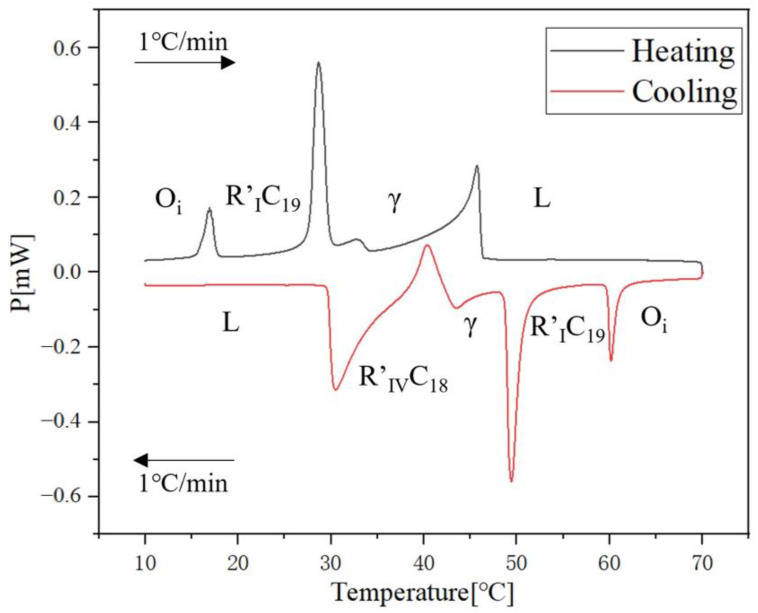
DSC analysis of Sample No. 11 (wt50% each for n-Nonadecane and 1-Octadecanol).

The specific phase transition for the heating and cooling procedure is shown in Figure 14. This result is proof of the peak for phase transition R’IV, which of 1-Octadecanol is not shown, also mentioned in the description for Figure 13 previously.

## 3. Materials and Methods

### 3.1. Mass Calculation

Twenty-one samples which had different ratio mixtures of n-Nonadecane and 1-Octadecanol (Produced by Sigma-Aldrich^®^, Leeds, UK) were created at the beginning of the experiment. The samples were differenced by 5% each. All the samples were thoroughly mixed by heating to 80 °C in the sample bottles on the electric heater and mixed by magnetic stirrer to confirm that they were in equilibrium. Then, the samples were crushed into powder with a mortar and pestle since all the analysis methods require fine powder for the analysis.

The desired weight size of each sample was around 30–40 mg.

The total molar weight is:

(1) $$M_{total}=\sum mole\%\times M$$

The weight percentage of each chemical is:

(2) $$Wt\%=\frac{mole\%\times M}{M_{total}}$$

The mass of each chemical for making the samples is:

(3) Mass(g)=Wt%×20

The compositions of samples (mol%) we prepared are listed in Table 3 and Table 4:

**Table 3:** The chemicals information of each sample.

Sample No.	Molar Fraction (C_18_OH/C_19_)	n-Nonadecane Mass (g)	1-Octadecanol Mass (g)	Total Mass (g)
1	0	0	0.03	0.03
2	5	0.001923	0.037107	0.03903
3	10	0.003846	0.035154	0.039
4	15	0.005769	0.033201	0.03897
5	20	0.007692	0.031248	0.03894
6	25	0.009615	0.029295	0.03891
7	30	0.011538	0.027342	0.03888
8	35	0.013461	0.025389	0.03885
9	40	0.015384	0.023436	0.03882
10	45	0.017307	0.021483	0.03879
11	50	0.01923	0.01953	0.03876
12	55	0.021153	0.017577	0.03873
13	60	0.023076	0.015624	0.0387
14	65	0.024999	0.013571	0.03857
15	70	0.026922	0.011718	0.03864
16	75	0.028845	0.009765	0.03861
17	80	0.030768	0.007812	0.03858
18	85	0.032691	0.005859	0.03855
19	90	0.034614	0.003906	0.03852
20	95	0.036537	0.001953	0.03849
21	100	0.03	0	0.03

### 3.2. Data Collection and Analysis

#### 3.2.1. DSC

The Differential Scanning Calorimetry (DSC) instrument was used to investigate the long-chain hydrocarbons’ thermal properties. These properties included the phase change enthalpy, melting and solidification temperature, and total enthalpy. For the purposes of the measurements, the instrument was calibrated using internal standards crucible made of aluminium. The DSC measurements were carried out in an inert N_2_ environment with a heating–cooling rate of 1 °C min^−1^ throughout a temperature range of 20 to 70 degrees Celsius, three cycles for each sample, and the weight of reference crucible was 49.00 mg.

The method of DSC used during this project was a heatwave-type, and the heating and cooling rate for major experiments was 1 °C per minute and a 3-time repeat. This step was designed to analyse the fusion and crystallisation process of the samples and using a different rate, which was 0.75 and 0.5 °C per minute for further analysis.

STAR^e^ software was used to analyse the collected DSC diagrams, displaying the Onset Temperature, End Set Temperature, and Peak Temperature, integrating the peaks, obtaining the area of each peak, and shading the peak area for further calculation.

#### 3.2.2. FTIR

Utilizing Fourier transform infrared (FTIR) spectroscopy, long-chain hydrocarbons were chemically characterised. Between 4000 and 400 cm^−1^, a NICOLET iS10 produced by Thermo-Scientific (Waltham, MA, USA) was utilised for the FTIR analyses. OriginLab^®^ (Version No.2022b) was used to plot the infrared spectra after obtaining the raw data from the FTIR instrument (Model No.: NICOLET iS10, Produced by Thermo-Scientific, Waltham, MA, USA). Moreover, we conducted an analysis of the wavenumber of the peaks and obtained different vibration types of each sample.

## 4. Discussions and Prospects

The Fourier transform infrared (FTIR) analysis, based on previous studies, provided valuable insights into the molecular dynamics of Organic Polymer Functional Adsorption Materials. The identification of peaks at specific wavenumbers, such as 750 cm^−1^ for the O-H bond bending, 1000 cm^−1^ for the C-O bond stretching, 1500 cm^−1^ for the C-H bond bending, and 3000 cm^−1^ for the saturated C-H stretching vibration absorption, offered a detailed understanding of the material’s composition. Additionally, the detection of a broad peak at 3300 cm^−1^, indicating O-H stretching vibration related to intermolecular hydrogen bonds, highlighted the complex molecular interactions within the binary system, akin to the intricate nature of plant cuticle waxes. Pascal S. [39] found that the synthesis pathway of plant epidermal wax can be divided into three steps: (1) de novo synthesis of C_16_ and C_18_ fatty acids; (2) C_16_ and C_18_ fatty acids extend to form waxy synthesis precursors, namely VLCFAs; (3) Waxy components such as alkanes, aldehydes, ketones, primary alcohols, secondary alcohols, and wax esters are synthesised by VLCFAs through acyl reduction and decarboxylation pathways. The above synthesis process is completed in plant epidermal cells. The results of FTIR-related research in this paper will play a supporting role in the synthetic waxy layer of plant epidermis.

Exploring the complexities of the 1-Octadecanol and n-Nonadecane binary system revealed a fascinating sequence of phase transitions, showcasing the dynamic structural changes of these compounds under varying conditions. 1-Octadecanol transitions from a liquid state to an R’IV phase before stabilizing in a monoclinic structure, while n-Nonadecane follows a unique transformation path from a liquid state to the R’I phase and eventually settling into an orthorhombic structure. These distinct molecular behaviours underscore the individuality of n-Nonadecane compared to 1-Octadecanol within the same binary system. Zhang [44] found that different cuticle waxes can form different and reversible wax modifications, thus adjusting the water transpiration and adsorption properties of the leaf cuticle in response to the degree of water change in the soil. Skamnioti [45] found that changes in cuticle permeability can affect plant disease resistance. Therefore, based on the phase transition path analysed in this paper, the plant epidermal wax layer modification can be artificially adjusted to improve the water transpiration and adsorption and disease resistance properties of plants.

Agriculture stands as one of the most crucial primary industries globally, serving as a fundamental pillar for sustaining basic livelihoods across all countries and regions. In the realm of plant biology, leaves play a vital role in almost all non-coniferous species, housing chloroplasts that harness sunlight for photosynthesis and cuticles that help regulate osmotic pressure and maintain optimal water levels within the leaves. Current research aims to explore the intricate relationship between plant cuticle wax and osmosis, shedding light on how plants endure various abiotic stresses like heat, drought, metals, cold, salt, and flooding. The mechanisms by which plants adapt to such stresses remain a topic of ongoing investigation. Furthermore, enhancing crop yields is a pressing global challenge, prompting scholars to delve deep into avenues for improvement through physical, chemical, biological, and geographical means. The outcomes of this project are poised to revolutionise crop performance, potentially through the optimisation of plant cuticle waxes using genetic engineering or similar techniques. By serving as a protective barrier for the plant epidermis, cuticle wax can also shield against growth-enhancing agrochemicals, offering insights for designing agrochemicals that can bypass this protective layer. Moreover, this project presents an opportunity to create a biomimetic plant surface through chemical processes, with potential applications in diverse fields like biology and beyond [46].

## Figures and Tables

**Table 1:** Comparison of Transition Enthalpy between the Experimental Results and the Mean Values of the Literature.

C_n_	Molar Mass (g/mol)	T_m_ (K)	ΔH_m_ (kJ/mol)	T_R-C_ (K)	ΔH_R-C_ (kJ/mol)	ΔH_total_ (kJ/mol)	Reference
18	270.49	330.3	-	328.00	-	-	Miquel Àngel, 2021 [37]
		328.2	44.742	321.78	15.756	60.498	This study
19	268.52	304.9	45.580	295.5	13.750	59.330	Dirand et al., 2002 [35]
		305.1	46.047	294.5	13.801	59.848	Cholakova et al., 2019 [38]
		303.6	31.417	294.2	9.134	40.551	This study

**Table 2:** Comparison of Different Compositions between 1-Octadecanol and n-Nonadecane.

Composition (C_18_OH %)	M_w_ (g/mol)	T_m_ (K)	ΔH_m_ (kJ/mol)	T_R-γ_ (K)	ΔH_R-γ_ (kJ/mol)	T_L-RI_ (K)	ΔH_L-RI_ (kJ/mol)	T_R-Oi_ (K)	ΔH_R-Oi_ (kJ/mol)	ΔH_total_ (kJ/mol)
0	268.5200	303.60	9.134	-	-	301.79	31.417	-	-	40.551
5	268.6185	305.02	7.211	-	-	301.89	31.093	-	-	38.304
10	268.7170	308.33	3.727	-	-	302.03	28.532	290.27	6.909	39.168
15	268.8155	316.75	4.449	-	-	301.71	27.107	290.18	6.804	38.36
20	268.9140	318.38	6.868	-	-	301.81	26.061	290.30	6.4619	39.391
25	269.0125	319.15	8.248	-	-	301.97	24.074	290.21	5.617	37.939
30	269.1110	319.62	9.368	-	-	301.86	22.662	290.11	5.253	37.283
35	269.2095	321.05	11.853	-	-	301.91	21.542	289.87	4.937	38.332
40	269.3080	321.55	13.024	-	-	301.84	21.203	289.69	4.551	38.778
45	269.4065	322.61	13.899	-	-	301.80	18.306	1289.83	3.483	35.688
50	269.5050	323.3	15.669	305.48	2.647	301.87	15.402	289.98	3.339	37.057
55	269.6035	324.42	17.565	307.68	4.341	302.02	13.270	290.07	2.879	38.055
60	269.7020	325.25	18.127	309.11	5.453	302.09	11.373	289.97	2.368	37.321
65	269.8005	325.62	19.431	310.47	6.904	302.07	10.279	289.78	1.983	38.597
70	269.8990	326.41	20.569	312.95	9.471	302.15	8.461	289.16	1.636	40.137
75	269.9975	326.55	19.872	313.68	9.080	302.14	5.989	289.91	1.237	36.178
80	270.0960	327.27	21.454	315.56	11.590	302.11	4.303	-	-	37.347
85	270.1945	328.26	21.983	317.07	13.839	302.04	3.132	-	-	38.954
90	270.2930	328.42	22.891	318.07	13.836	-	-	-	-	36.727
95	270.3915	329.6	26.114	320.77	15.407	-	-	-	-	41.521
100	270.4900	329.86	44.742	321.85	15.756	-	-	-	-	60.498

**Table 4:** The crucible weight and sample weight for reproducibility (in crucible).

Sample No.	Molar Fraction (C_18_OH/C_19_)	Crucible Weight (Empty)/mg	Crucible Weight (Full)/mg	Sample Weight (mg)
Re-1 (1)	0	48.38	58.07	9.69
Re-1 (11)	50	48.63	58.54	9.91
Re-1 (21)	100	48.43	56.57	8.14
Re-2 (1)	0	48.26	56.19	7.93
Re-2 (11)	50	48.59	55.06	6.47
Re-2 (21)	100	49.19	59.61	7.93

## Data Availability

All the data can be collected by contacting wentao_guo@ustb.edu.cn.
